# NAD+ METABOLISM IN MITOCHONDRIA AND MICROBES

**DOI:** 10.1093/geroni/igad104.0597

**Published:** 2023-12-21

**Authors:** Joseph Baur

**Affiliations:** Perelman School of Medicine at the University of Pennsylvania, Philadelphia, Pennsylvania, United States

## Abstract

Nicotinamide adenine dinucleotide (NAD+) is a redox cofactor essential to all living organisms. NAD+ levels fall with age or disease and rise with exercise or caloric restriction. Supplemental NAD+ precursors have beneficial effects on health in rodent models, renewing interest in this molecule and the enzymes that require it. Although NAD+ is present in the mitochondrial matrix and critical to the function of the organelle, the source of mitochondrial NAD+ was only recently elucidated. We identified SLC25A51 as the carrier that is primarily responsible for this activity in cultured cells. Here we show that, like in cells, SLC25A51 is important for establishing the mitochondrial NAD+ pool in vivo. Shifting NAD+ into hepatic mitochondria is sufficient to replicate the effects of supplemental NAD+ precursors on liver regeneration. Thus, the mitochondrial NAD+ pool appears to be responsible for some of the benefits of increasing whole-body NAD+. In a separate series of studies, we have used heavy isotope tracing strategies to show that the popular supplement nicotinamide riboside is metabolized by the microbiome when administered orally to mice. Further studies revealed a surprising role for the microbiome in normal NAD+ metabolism. Host-derived nicotinamide is released into the gut lumen and processed to generate microbial NAD+ as well as nicotinic acid that is reabsorbed by the host. These findings reveal a previously unappreciated cycle by which NAD+ precursors are shared between the host and microbes.

